# Transcription factor NFAT5 contributes to the glycolytic phenotype rewiring and pancreatic cancer progression via transcription of PGK1

**DOI:** 10.1038/s41419-019-2072-5

**Published:** 2019-12-11

**Authors:** Yongsheng Jiang, Ruizhe He, Yuhong Jiang, Dejun Liu, Lingye Tao, Minwei Yang, Chaoyi Lin, Yang Shen, Xueliang Fu, Jianyu Yang, Jiao Li, Yanmiao Huo, Rong Hua, Wei Liu, Junfeng Zhang, Baiyong Shen, Zhigang Zhang, Yongwei Sun

**Affiliations:** 1 Department of Biliary-Pancreatic Surgery, Ren Ji Hospital, School of Medicine, Shanghai Jiao Tong University, Shanghai, P. R. China; 2 Department of General Surgery, Rui Jin Hospital, School of Medicine, Shanghai Jiao Tong University, Shanghai, P. R. China; 3 Qingdao Women and Children’s Hospital, Qingdao, China; 4 Department of Hepatobiliary Pancreas Surgery, Shanghai East Hospital, Tong Ji University School of Medicine, Shanghai, P. R. China; 5 State Key Laboratory of Oncogenes and Related Genes, Shanghai Cancer Institute, Ren Ji Hospital, School of Medicine, Shanghai Jiao Tong University, Shanghai, P. R. China

**Keywords:** Cancer metabolism, Pancreatic cancer, Oncogenes

## Abstract

Hypoxia and the hypovascular tumor microenvironment are major hallmarks of pancreatic ductal adenocarcinoma (PDAC), in which glycolysis is of great importance to tumor survival and proliferation. There is little research regarding the role of Nuclear Factor of Activated T Cells 5 (NFAT5) in relation to carcinoma. Here, we explored the impact of NFAT5 on the biological behavior of PDAC and the underlying mechanism. We demonstrated that NFAT5 was highly expressed in PDAC and was related to poorer prognosis. Knockdown of NFAT5 lead to impaired proliferation of tumor cells caused by an aberrant Warburg effect. Mechanically, phosphoglycerate kinase 1 (PGK-1), which is the first enzyme generating ATP in glycolysis, was verified as a target gene of NFAT5. Over-expression of PGK1 compromised the aberrant oncological behavior caused by knockdown of NFAT5 both in vitro and in vivo. Clinical samples underwent positron emission tomography-computed tomography (PET-CT) examination and KrasG12D/+/Trp53R172H/+/Pdx1-Cre (KPC) mice were collected to support our conclusion.

## Introduction

Pancreatic cancer is one of the most aggressive and lethal malignant tumors, with a continuously rising mortality^1,2^. Pancreatic cancer ranks fourth among the fatal malignant tumors in western countries and sixth in China; its 5-year survival rate is only 7% or lower^2–4^. Because of its unique anatomical location and structure, as well as its physiological characteristics, it has no obvious symptoms at the early stage of onset and it is consequently quite difficult to diagnose. For the purpose of enhancing the survival rate and improving the life quality of patients, biological and genetic studies of pancreatic cancer are imperative.

There is increasing evidence of the pro-tumor role of the NFAT (Nuclear Factor of Activated T cell) family in multiple kinds of malignancies. The NFAT family consists of five transcription factors including NFATc1, NFATc2, NFATc3, NFATc4, and NFAT5^5^, most of which have been well-studied in development processes. For instance, NFATc1 is able to transform preadipocyte cells via the JAK-Stat3 pathway, and NFATc3 can contribute to malignant melanoma progression. However, the specific role of these transcription factors in pancreatic ductal adenocarcinoma (PDAC) has not been well-studied. Here, we found that all five transcription factors are highly expressed in tumor tissue, and the expression of NFAT5 is negatively associated with prognosis. NFAT5 (Nuclear Factor Of Activated T Cells 5) was first recognized as a tonicity-regulated transcription factor (Ton/EBP) that regulates the expression of genes involved in osmotic stress^6,7^. Recent studies revealed the potential relationship between NFAT5 and several cancers. In melanoma, NFAT5 was shown to react in the cell cycle and play a driving role in the development and carcinoma metastasis^8^. However, in hepatocellular carcinoma (HCC), NFAT5 was identified as a tumor suppressor gene^9,10^. These findings indicated that NFAT5 has a controversial role in cancer, and the exact role of NFAT5 in pancreatic cancer has not been reported.

In our study, we demonstrated that NFAT5 contributes to glycolytic phenotype rewiring and pancreatic cancer progression via transcription of PGK1. Glycolysis is active in cancer cells even when oxygen is sufficient. This metabolic characteristic of aerobic glycolysis is known as the Warburg effect^11^. Because pancreatic cancer is a hypovascular tumor^12^, it is essential to investigate the role of the Warburg effect in pancreatic cancer. The Warburg effect has been extensively studied, but its exact essence is still unclear, which hinders the exploration of its therapeutic targets^13^.

Our study demonstrated that NFAT5 is upregulated in pancreatic cancer cells, and NFAT5 facilitates PDAC cell survival via contributing to the Warburg effect by transcription of PGK1.

## Materials and methods

### Cell culture and reagents

Human pancreatic cancer AsPC-1, BxPC-3, CAPAN-1, CFPAC-1, PANC-1, and SW1990 cell lines were purchased from the Cell Bank of the Chinese Academy of Sciences (Shanghai, China). AsPC-1 and BxPC-3 cells were cultured in RPMI 1640 culture medium with 10% fetal bovine serum (FBS) and 1% penicillin/streptomycin (P/S), while the others were cultured in Dulbecco’s modified Eagle’s medium (DMEM) with 10% FBS and 1% P/S. The culture conditions were 37 °C and a 5% CO_2_ atmosphere. For studies in hypoxia, cells were grown at 37 °C in an atmosphere of 1% O_2_. For the cellular function assay, all PDAC cells were cultured in medium with 5 mM glucose and 2 mM L-glutamine in the absence of FBS. Experiments in Figs. 4c–f, 5c and 7b–f were performed with cells cultured in hypoxia condition to better simulate the hypovascular tumor microenvironment in PDAC. Cell function assays performed in non-hypoxia conditions were shown in Figs. S1–S3.

### Knockdown and overexpression assay

The lentivirus against NFAT5 was purchased from Gene Pharma (Shanghai, China), and the sequences targeting NFAT5 were: sh-1, 5′-GGCACACAGUCUUGUACAUTT-3′ (sense), 5′-AUGUACAAGACUGUGUGCCTT-3′ (antisense) and sh-2, 5′-CACUGAGGUACCUCGUAA-ATT-3′ (sense), 5′-UUUACGAGGUACCUCAGUGTT-3′ (antisense). LV3-pGLV-h1-GFP-puro vector was used for NFAT5 knockdown experiments. For transducing lentivirus, cells were cultured in a six-well plate, and 200 μl lentivirus suspension was added in the presence of 5 μg/ml polybrene (Gene Pharma, Shanghai, China). Forty-eight hours after transduction, 5 μg/ml puromycin was added into culture medium for stable cell line screening. For the PGK1 overexpression assay, PGK1-expressing plasmid was transfected into the cells in the presence of Lipofectamine 3000 (Thermo Fisher Scientific).

### Human PDAC samples, patient information, and tissue microarray (TMA)

Primary pancreatic cancer tissues were obtained from pancreatic cancer patients treated at Shanghai Jiao Tong University Affiliated Renji Hospital after receiving informed consent. TMA containing 311 pairs of PDAC specimens and corresponding noncancerous tissues were obtained from Ren Ji Hospital from January 2002 to June 2015. For all samples from PDAC patients, clinical information was available. The pathological information was retrieved from the Pathology Department, while the positron emission tomography-computed tomography (PET-CT) images were retrieved from the Nuclear Medicine Department. The follow-up time was calculated from the date of surgery to pancreatic cancer-related death. All the patients were provided with written informed consent before enrolment, and the study was approved by the Research Ethics Committee of Ren Ji Hospital, School of Medicine, Shanghai Jiao Tong University.

### Quantitative real-time PCR

Total RNA was extracted using Trizol reagent (Takara, Japan) according to the manufacturer instructions. cDNA was synthesized using a PrimeScript RT Reagent Kit (Takara, Japan) in accordance with the protocol of the manufacturer. A StepOne Real-Time PCR System (Applied Biosystems, Grand Island, NY, USA) was applied to detect the expression level of the target gene using the SYBR Premix Ex Taq II (Takara, Japan), and β-actin acted as an internal control. The data were assessed using the 2-ΔΔCt method. Primer sequences are listed as follows: NFAT5 forward, 5′-GGGTCAAACGACGAGATTGTG-3′, reverse, 5′-GTCCGTGGTAAGCTGAGAAAG-3′; GLUT1 forward, 5′-ATTGGCTCCGGTATCGTCAAC-3′, reverse, 5′-GCTCAGATAGGACATCCAGGGTA-3′; HK2 forward, 5′-AGCCCTTTCTCCATCTCCTT-3′, reverse, 5′-GCTTGCCTACTTCTTCACGG-3′; GPI1 forward, 5′-CAAGGACCGCTTCAACCACTT-3′, reverse, 5′-CCAGGATGGGTGTGTTTGA-CC-3′; PFKL forward, 5′-GGTGCCAAAGTCTTCCTCAT-3′, reverse, 5′-GATGATGTTG-GAGACGCTCA-3′; ALDOA forward, 5′-AACTTTCCTCTGCCTAGCCC-3′, reverse, 5′-GTACAGGCACAGTCGCAGAG-3′; TPI1 forward, 5′-AGCTCATCGGCACTCTGAAC-3′, reverse, 5′-CCACAGCAATCTTGGGATCT-3′; PGK2 forward, 5′-AAACTGGATGTTAGA-GGGAAGCG-3′, reverse, 5′-GGCCGACCTAGATGACTCATAAG-3′; PGAM2 forward, 5′-AGAAGCACCCCTACTACAACTC-3′, reverse, 5′-TCTGGGGAACAA-TCTCCTCGT-3′; ENO1 forward, 5′-GCCGTGAACGAGAAGTCCTG-3′, reverse, 5′-ACGCCTGAA-GAGACTCGGT-3′; PKM2 forward, 5′-ATGTCGAAGCCCCATAGTGAA-3′, reverse, 5′-TGGGTGGTGAATCAATGTCCA-3′; LDHA forward, 5′-ATGGCAACTCTAA-AGGATCAGC-3′, reverse, 5′-CCAACCCCAACAACTGTAATCT-3′; PDK1 forward, 5′-CTGTGATACGGATCAGAAACCG-3′, reverse, 5′-TCCACCAAACAATAAAGAGTGCT-3′; PGK1 forward, 5′-GAACAAGGTTAAAGCCGAGCC-3′, reverse, 5′-GTGGCAGATTG-ACTCCTACCA-3′; MET forward, 5′-GGTTCACTGCATAT-TCTCCCC-3′, reverse, 5′-ACCATCTT-TCGTTTCCTTTAGCC-3′; GAPDH forward, 5′-GCATTGCCCTCAACG-ACCAC-3′, reverse: 5′-CCAC-CACCCTGTTGCTGTAG-3′ and β-actin, forward 5′-ACTCGTCATACTCCTGCT-3′, reverse 5′-GAAACTACCTTCAACTCC-3′

### Western blotting (WB)

The WB was conducted as reported previously^14^. Antibodies used were mouse anti-GAPDH (1:10,000, Abcam, ab8245), rabbit anti-NFAT5 (1:1000, Abcam, ab226308), and rabbit anti-PGK1 (1:500, Abcam, ab38007).

### Immunohistochemistry (IHC)

IHC was performed as previously described^14^. Anti-NFAT5 (1:100, Abcam, ab226308), PGK1 (1:100, Abcam, ab38007), and anti-PCNA (1:500, Cell Signaling Technology, #13110) antibodies were used. Tumor tissues were scored according to the percentage of stained area (0 = 0%, 1 = 1–10%, 2 = 11–50%, 3 = 51–80%, and 4 = 81–100%) and darkness of the nuclei or cytoplasm staining (0 = no staining, 1 = weak staining, 2 = moderate staining, and 3 = strong staining). Final scores were determined by multiplying the 2 numbers mentioned above (“negative” for a score of 0, “weak” for a score of 1–4, “moderate” for a score of 5–8, and “positive” for a score of 9–12). The high-expression group had scores greater than 5, while the low-expression group scores were ≤5.

### CCK-8 and colony formation assays

Cell viability was measured using a Cell Counting Kit-8 (CCK-8, Dojindo Molecular Technologies, Japan). Cells were collected after stable transfection for 72 h, and then they were seeded in plates (96-well) at a density of 2 × 10^4^ cells/ml. CCK-8 (10 μl/well) reagent was added to each well at 0, 1, 2, 3, 4, and 5 days. The absorbance at each wavelength of 450 nm was measured by an automatic enzyme-linked immune detector after 1 h of incubation. The cell growth curve was plotted with time on the horizontal axis and the OD value on the vertical axis. The experiment was repeated three times.

In the colony formation test, target cells were seeded into six-well plates. Two weeks later, the colonies were fixed with polyformaldehyde and stained with 0.5% (w/v) crystal violet. Photographs were taken and cell counts were calculated.

### Seahorse analyses

The extracellular acidification rate (ECAR) and oxygen consumption rate (OCR) of the cells was assessed using the Seahorse XF96 Flux Analyser (Seahorse Bioscience, Agilent). In brief, 1 × 10^4^ cancer cells were seeded in an incubation plate as the protocol indicated. Cells were cultured at 37 °C overnight for adhesion. Before detection, culture medium was replaced with assay media. The glycolytic stress test kit (Seahorse Cat. #103020-100) and mitochondrial stress test kit (Seahorse Cat. #103015-100) were purchased for ECAR and OCR detection, respectively. The assays were performed using the manufacturer protocols.

### Glucose and lactate measurement

To perform the glucose consumption rate and lactate production assays, cells were cultured in a six-well plate for 24 h. The supernatants were then collected for further detection. The Amplex® Red Glucose/Glucose Oxidase Assay Kit (Invitrogen, Cat. # A22189) and Lactate Assay Kit (BioVision, Cat. # ABIN411683) were purchased for each assay.

### PDAC transgenic model and orthotopic xenograft model

The PDAC transgenic model and orthotopic xenograft model were utilized, as reported previously^15^.

### Promoter activity analysis by dual luciferase assay

Promoters of human PGK1 were cloned into pGL4.10-Basic vector. Cells were seeded in 96-well plates at a density of 1.2 × 10^5^ cells per well. The cells were transfected with vectors, either expressing wild-type or mutant PGK1 promoter using Lipofectamine 3000 (Thermo Fisher Scientific). Next, the cells were assayed for both firefly and Renilla luciferase activities using a dual luciferase system (Promega, Madison, WI, USA), as described in the manufacturer’s protocol. The experiments were performed in triplicate and repeated three times. The data are presented as the fold-change relative to the control group.

### Statistics

Data are presented as the means ± SD. Statistical analyses were performed using SPSS 22.0 for Windows (IBM). Cumulative survival time was calculated by the Kaplan–Meier method and analyzed by the log-rank test. Correlation of NFAT5 expression with categorical clinical variables in patients with PDAC was evaluated by *χ*2 test or Fisher’s exact test. Univariate and multivariate Cox regression analyses were performed to identify the factors that had a significant influence on survival by Cox proportional hazards model. The student’s *t*-test or one-way ANOVA was used for comparison between groups. Values of *P* < 0.05 were considered statistically significant.

## Results

### Oncogene screening of NFAT transcription factor family in PDAC

**Fig. 1:**
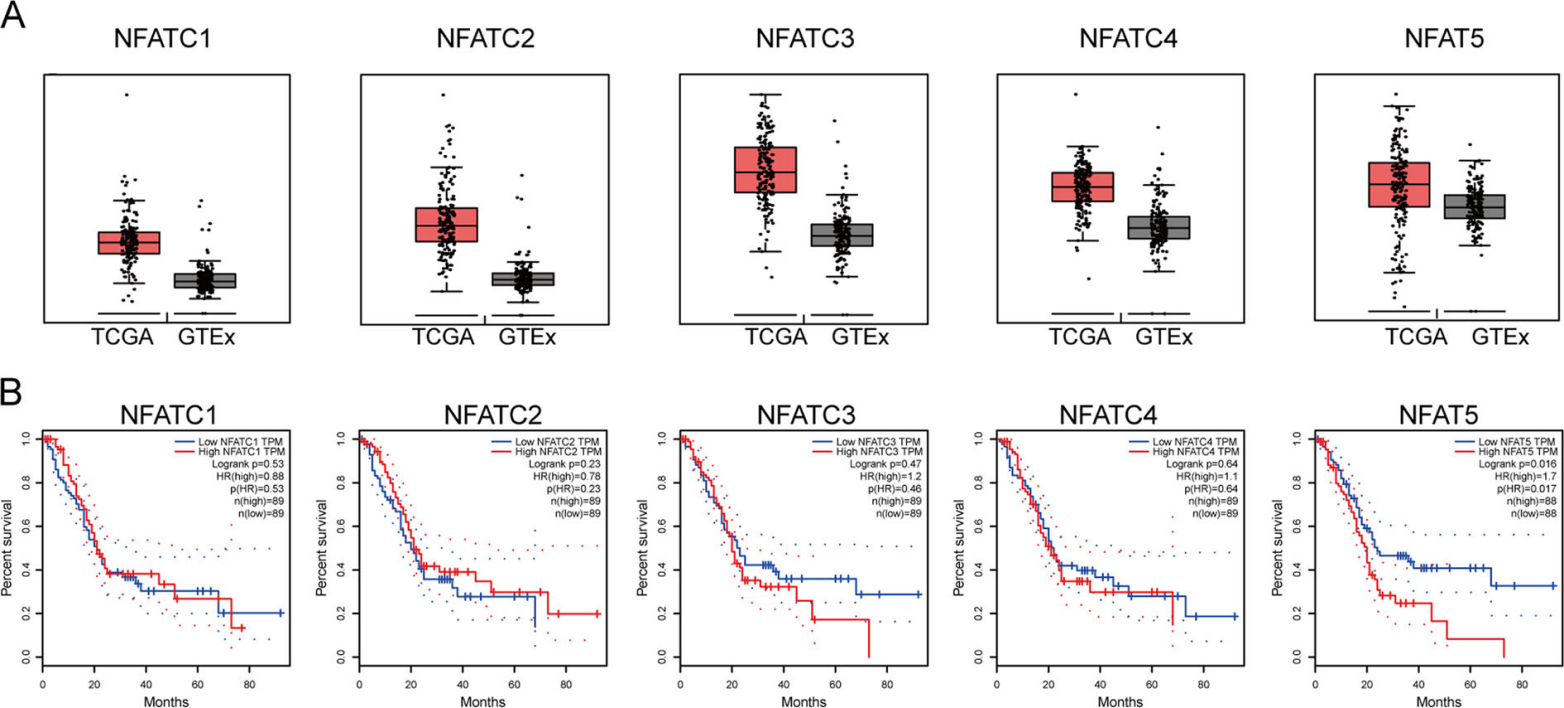
Screening process for NFAT5. **a** Five members, NFATC1, NFATC2, NFATC3, NFATC4, and NFAT5, of the NFAT family were highly expressed in the tumor tissue in TCGA database compared to the normal tissue in GTEx database. **b** Kaplan–Meier analyses of the prognostic value of NFATs based on the TCGA expression data.

**Fig. 2:**
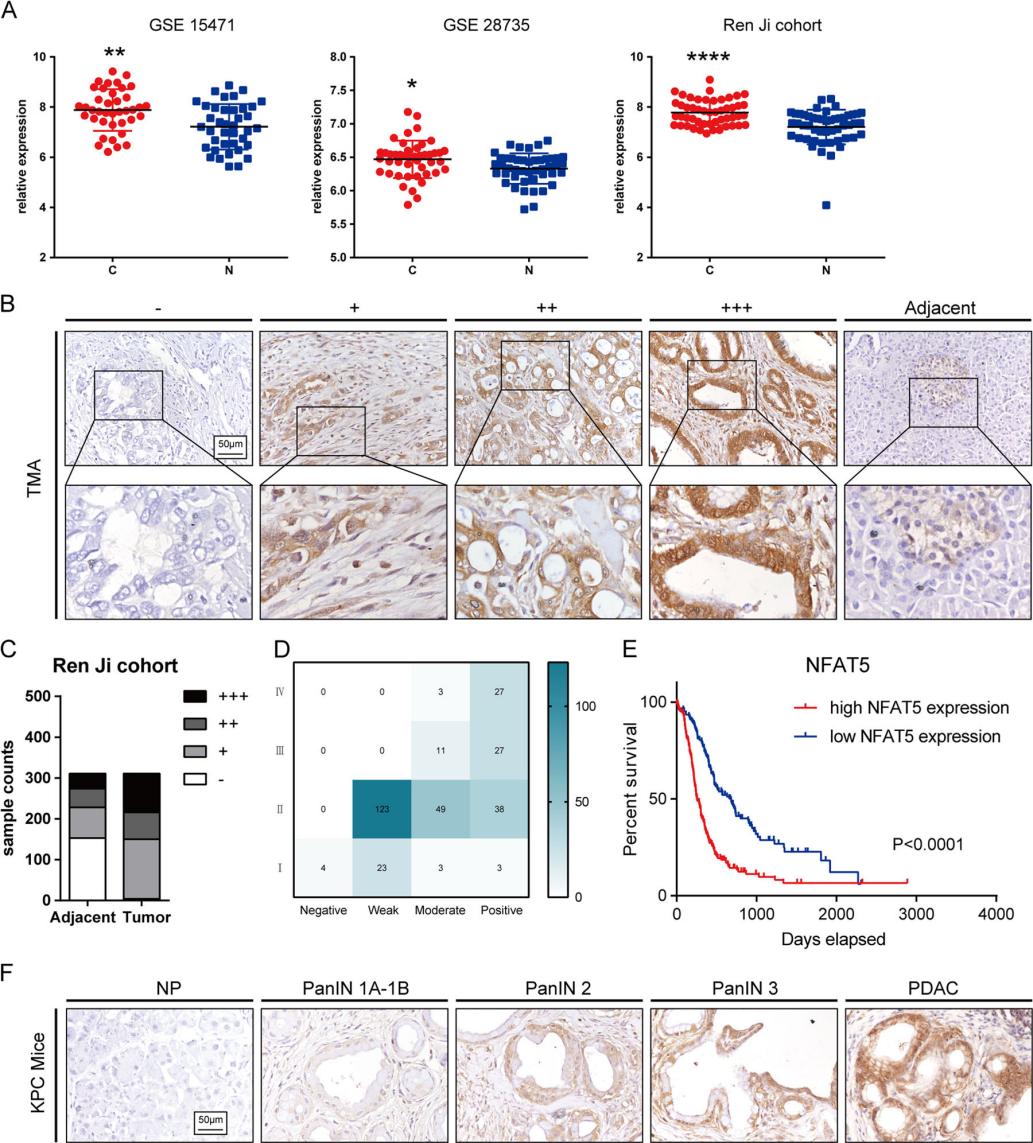
High NFAT5 expression was linked to poor prognosis in PDAC. **a** The NFAT5 expression of tumor tissues and paired non-tumor tissues in the GSE15471, GSE28735, and Ren Ji cohort. **b** Standard immunohistochemical scoring pictures of NFAT5 expression in 311 pancreatic cancer tumors and adjacent normal tissues. **c** Respective sample counts in tumor tissues and adjacent tissues. **d** Heatmap shows the counted numbers of four levels of NFAT expression and its correlation with TNM classification according to the IHC. **e** Kaplan–Meier analysis of overall survival rate related to the expression of NFAT5 expression in 311 cases based on the Renji patients. **f** Standard immunohistochemical pictures in different stages of PDAC progression in KPC mice. *P < 0.05, **P < 0.01, ****P < 0.0001.

To screen for the potential oncogene in the NFAT family, The Cancer Genome Atlas (TCGA) and The Genotype-Tissue Expression (GTEx) datasets were collected and analyzed. As shown in Fig. 1a, NFATC1, NFATC2, NFATC3, NFATC4, and NFAT5 were all highly expressed in the tumor tissue of the TCGA dataset compared with normal tissue of the GTEx dataset. However, in the TCGA dataset, only increased NFAT5 predicted poor prognosis (Fig. 1b), so we further investigated its role in PDAC. We first examined the NFAT5 expression in three Gene Expression Omnibus (GEO) datasets including our own Renji cohort. The results showed that NFAT5 manifested a higher expressed pattern compared with adjacent noncancerous tissue in all three datasets (Fig. 2a). We then validated our results on the protein level by performing IHC staining in a Renji TMA containing 311 pathologist-verified PDAC specimens with paired corresponding adjacent pancreatic tissue. The expressions were scored based on staining intensity and area (Fig. 2b). As shown in Fig. 2c–e, tumor tissue manifested a higher staining score, and patients with increased NFAT5 had a poorer prognosis. We then performed Chi-square analysis to evaluate the relationship between the expression of NFAT5 and clinicopathologic features of samples in the TMA cohort. The result showed that NFAT5 was significantly correlated with tumor size, tumor differentiation, lymph node metastasis, distant metastasis, and TNM stage (Table 1). We also used univariate and multivariate analysis to identify NFAT5 expression as an independent risk factor for PDAC (Table 2). We also detected the expression pattern of NFAT5 in KrasG12D/+/Trp-53R172H/+/Pdx1-Cre (KPC) mice. As shown in Fig. 2f, the staining of NFAT5 is positively correlated with the progression of PDAC. These data suggest that NFAT5 may play a role as an oncogene in PDAC.

**Table 1:** Correlation between NFAT5 expression and clinicopathologic factors.

Factors	Expression of NFAT5		*P* value
	Low (*n* = 150)	High (*n* = 161)	
Age (y)			
<65	72 (48%)	73 (45.3%)	0.639
≥65	78 (52%)	88 (54.7)	
Sex			
Male	86 (57.3%)	90 (55.9%)	0.799
Female	64 (42.7%)	71 (44.1%)	
Tumor size			
<2 cm	26 (17.3%)	11 (6.8%)	0.004 (***)
≥2 cm	124 (82.7%)	150 (93.2%)	
Tumor differentiation			
Moderate/poor	102 (68.0%)	92 (57.1%)	0.048 (*)
Well	48 (32.0%)	69 (42.9%)	
Lymph node metastasis			
Absent	113 (75.3%)	82 (50.9%)	<0.0001 (****)
Present	37 (24.7%)	79 (49.1%)	
Distant metastasis			
Absent	150 (100.0%)	131 (81.4%)	<0.0001 (****)
Present	0 (0.0%)	30 (18.6%)	
TNM stage			
I/II	150 (100.0%)	93 (57.8%)	<0.0001 (****)
III/IV	0 (0.0%)	68 (42.2%)	
Nerve invasion			
Absent	88 (58.7%)	77 (47.8%)	0.056
Present	62 (41.3%)	84 (52.2%)	

*p < 0.05, ***p < 0.01, ****p < 0.0001

**Table 2:** Univariate and Multivariate Cox regression analysis of potential prognostic factors in gastric cancer.

Factors	Univariate		Multivariate	
	HR (95% CI)	*P* value	HR (95% CI)	*P* value
Age	1.314 (1.000–1.727)	**0.05**	1.467 (1.103–1.951)	**0.008**
Gender	0.861 (0.655–1.132)	0.861		
Tumor size	1.682 (1.070–2.644)	**0.024**	1.240 (0.780–1.969)	0.363
Tumor differentiation	1.682 (1.274–2.222)	<0.0001 (****)	1.392 (1.043–1.857)	0.025 (*)
Lymph node metastasis	1.686 (1.279–2.223)	<0.0001 (****)	1.504 (1.120–2.021)	0.007 (***)
Distant metastasis	1.858 (1.201–2.874)	<0.005 (***)	1.227 (0.688–2.185)	0.488
TNM stage	1.827 (1.326–2.518)	<0.0001 (****)	1.003 (0.645–1.560)	0.991
Nerve invasion	1.147 (0.876–1.502)	0.319		
NFAT5 experssion	2.410 (1.821–3.190)	<0.0001 (****)	2.031 (1.474–2.798)	<0.0001 (****)

*p < 0.05, ***p < 0.01, ****p < 0.0001

### Genetic inhibition of NFAT5 suppresses PDAC cell growth both in vivo and in vitro

**Fig. 3:**
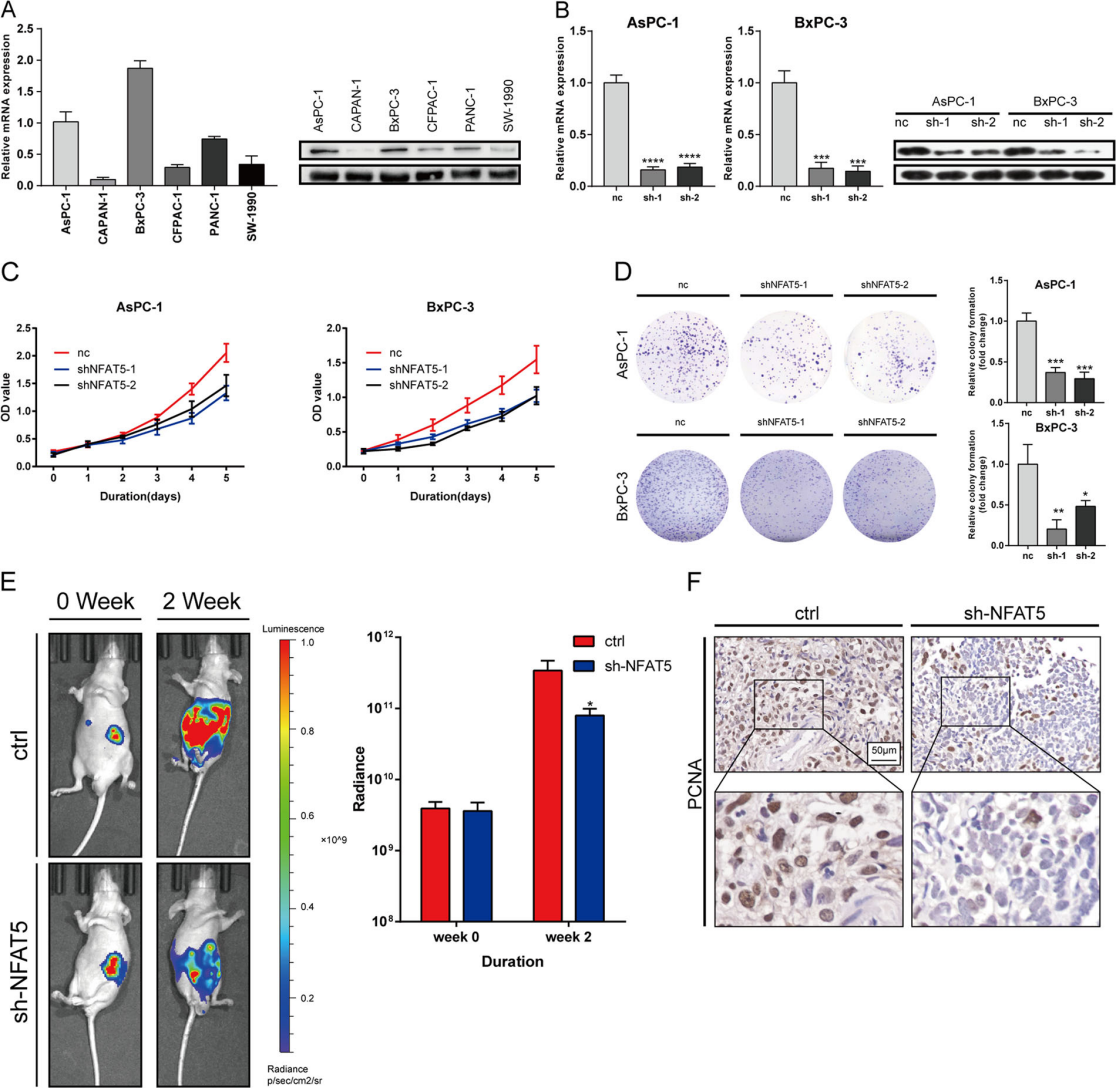
Genetic inhibition of NFAT5 suppresses PDAC cell growth both in vivo and in vitro. **a** Relative mRNA and protein levels of NFAT5 in six pancreatic cancer cell lines. **b** The mRNA and protein expression of NFAT5 in NFAT5 knockdown AsPC-1 and BxPC-3 treated with shRNA. **c** The CCK-8 assay showed that NFAT5 knockdown in AsPC-3 and BxPC-3 cells resulted in suppressed proliferation capability. **d** The colony formation ability was restrained in NFAT5 knockdown PDAC cells. **e** Representative bioluminescence photograph of mice orthotopically implanted with luciferase-expressing AsPC-1 cells with or without NFAT5 knockdown. **f** IHC staining of PCNA in mice orthotopically implanted tissue. *P < 0.05, **P < 0.01, ****P < 0.0001.

To study the effect of NFAT5 on PDAC, we examined the expression level of NFAT5 in six pancreatic cancer cell lines both at the mRNA and protein level, at which AsPC-1 and BxPC-3 exhibit the highest expression (Fig. 3a). Two cell lines were selected for knockdown assay, which was assessed at the mRNA and protein level (Fig. 3b). We then performed CCK8 assays and colony formation assays to evaluate the pro–proliferation effect of NFAT5 on tumor cells (Fig. 3c, d). The orthotopic xenograft model was established to test the effect of NFAT5 in vivo. As shown in Fig. 3e, mice injected with NFAT5 knockdown cell lines (AsPC-1) occupied lower bioluminescent emission compared with the control group (AsPC-1). The IHC result for specimens also indicated that the proliferation of the control group was stronger than that of the NFAT5 knockdown group (Fig. 3f).

### NFAT5 facilitates PDAC cell survival via contributing to the Warburg effect

**Fig. 4:**
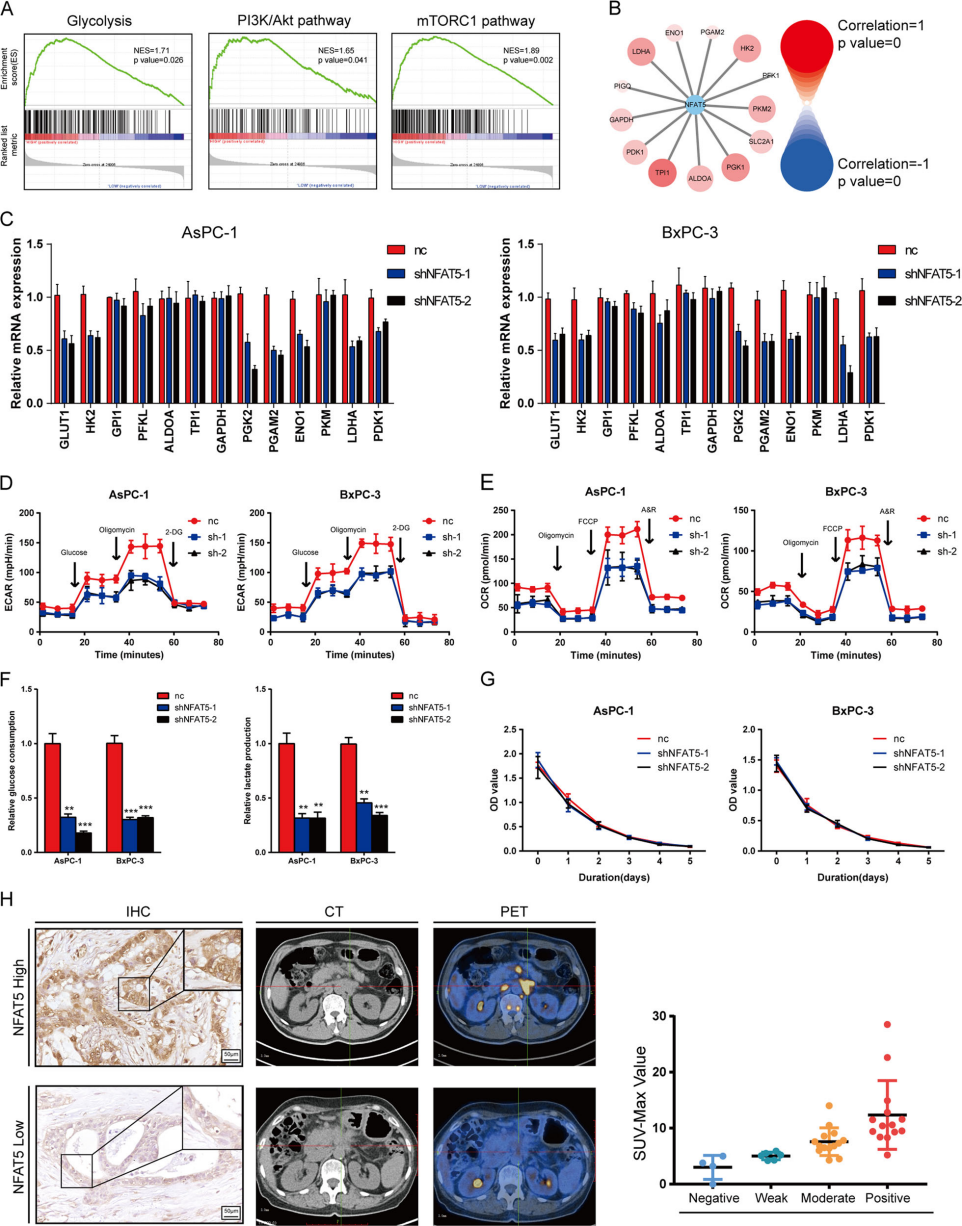
NFAT5 promoted PDAC cell growth by enhancing the Warburg effect. **a** Gene set enrichment analysis (GSEA) of the NFAT5 high-expression group and the low-expression group using hallmark gene sets showed the relationship between NFAT5 and three datasets closely related to the Warburg effect. NES, normalized enrichment score. **b** The correlation between NFAT5 and glycolytic enzymes. **c** Relative mRNA levels of glycolysis-related genes of PDAC with or without NFAT5 knockdown. **d**, **e** Glycolytic function and mitochondrial stress test of NFAT5 knockdown AsPC-1 and BxPC-3 cells treated with shRNA were measured by extracellular acidification rate (**d**) and oxygen consumption rate (**e**), respectively. **f** Relative glucose consumption and lactate production in normal and NFAT5 knockdown AsPC-1 and BxPC-3 cell lines. **g** NFAT5 knockdown AsPC-1 and BxPC-3 cell proliferation capability indicated no difference after glucose was replaced with galactose compared to normal AsPC-1 and BxPC-3 cells. **h** Typical immunohistochemical images of NFAT5 low and high-expression group along with their CT and PET-CT images. The relationship between 4 NFAT5 expression groups and SUV-Max Values was counted. Assays shown in Fig. 4c, f were performed in hypoxia condition. *P < 0.05, **P < 0.01, ****P < 0.0001.

To understand how NFAT5 influences pancreatic tumor cell growth, we first performed Geneset Enrichment Analysis (GSEA) by dividing samples into two groups based on NFAT5 expression in the Renji cohort. As shown in Fig. 4a, three datasets closely related to the Warburg effect (glycolysis, PI3K/AKT/mTOR signaling, and mTORC1 signaling) were enriched in the NFAT5 high-expression group, indicating that NFAT5 may have an impact on glucose metabolism in pancreatic cancer. We then assessed the correlation between NFAT5 and several key enzymes in the Warburg effect in datasets. As shown in Fig. 4b, almost all enzymes are positively correlated with NFAT5, suggesting that NFAT5 upregulates the Warburg effect in PDAC. To further validate the underlying reprogramed glucose metabolism modulated by NFAT5, we performed qPCR to investigate the effect of NFAT5 on glycolysis-related genes and found that knockdown of NFAT5 results in impaired expression of HK2, PGK1, and LDHA (Fig. 4c). Cells were cultured in hypoxia condition to simulate the hypovascular tumor microenvironment in PDAC. The extracellular acid rate (ECAR) and OCR of PDAC cells were measured by the Seahorse XF96 Flux Analyzer. The result showed that knockdown of NFAT5 significantly decreased both ECAR and OCR in two cell lines (Fig. 4d, e), indicating that NFAT5 plays a contributing role in glucose metabolism in PDAC. In addition, knockdown of NFAT5 also led to a marked decrease in glucose uptake and extracellular lactate levels in both AsPC-1 and BxPC-3 cell lines (Fig. 4f). We then aimed to investigate whether the effect of NFAT5 on PDAC glucose metabolism could facilitate the cell survival. To test our hypothesis, we replaced the glucose in culture medium with galactose, which blocks the glucose flux to eliminate the Warburg effect on cellular function. Just as anticipated, reduced glucose flux greatly compromised the pro-survival effect of NFAT5 (Fig. 4g). Then, we tested the expression status of NFAT5 by IHC staining and analyzed its correlation with 18F-FDG uptake in 39 pancreatic cancer patients who received PET-CT examination to validate our results in clinical samples. The results demonstrated that higher NFAT5 expression was accompanied with a higher standard uptake value (SUV-Max) (Fig. 4h). These data demonstrate that NFAT5 facilitates PDAC cell survival via contributing to the Warburg effect.

### PGK1 is a direct target gene of NFAT5

**Fig. 5:**
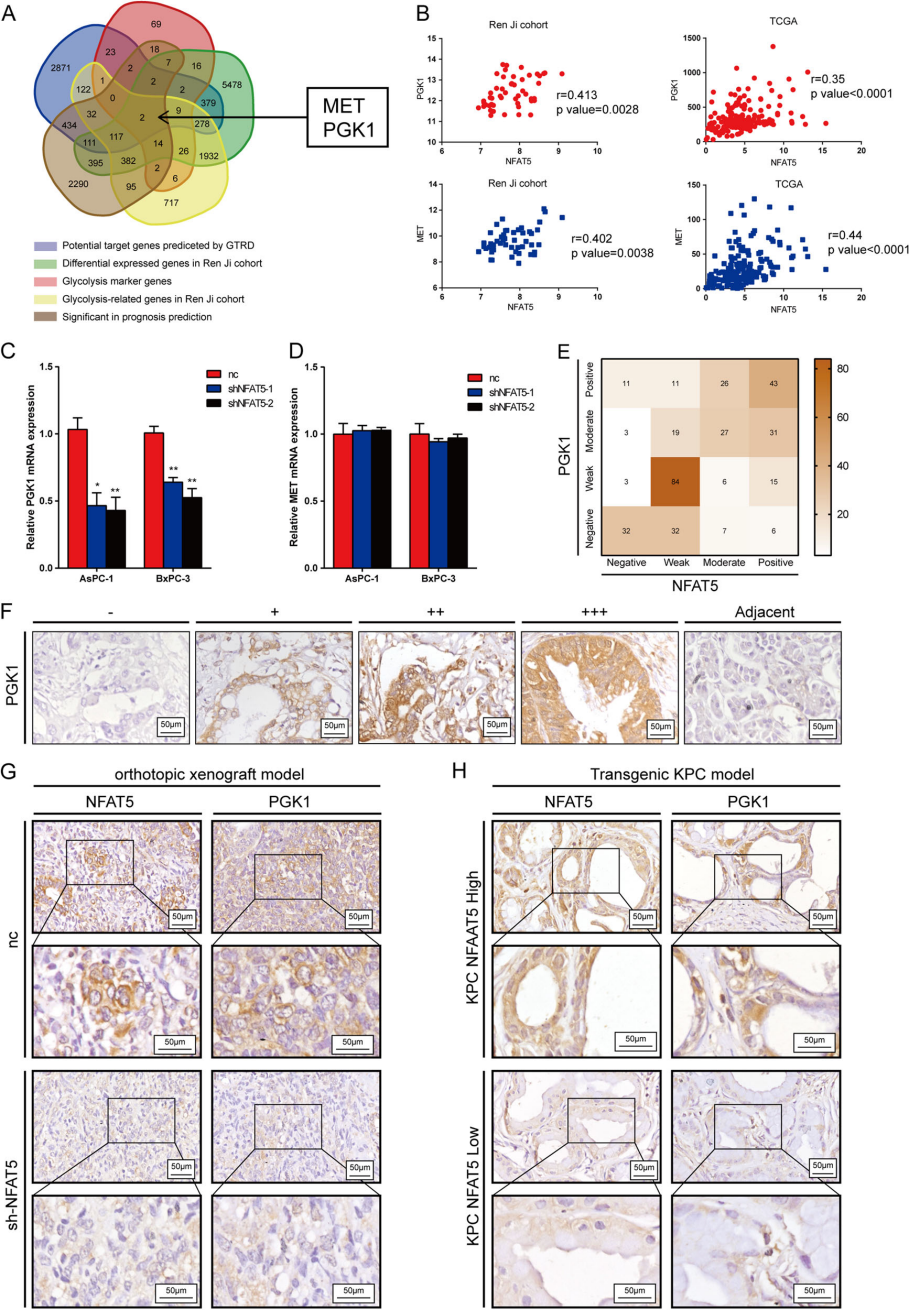
PGK1 is a direct target gene of NFAT5. **a** Venn diagram showing the intersection of five gene sets. **b** The relevance of NFAT5 with PGK1 and MET is displayed in the Renji cohort and TCGA database. **c**, **d** Relative PGK1 and MET mRNA expression in the control and NFAT5 knockdown PDAC cell lines. **e** Heatmap showing the counted numbers of four levels of PGK1 expression and its correlation with NFAT5. **f** Standard immunohistochemical scoring pictures of PGK1 expression in 311 pancreatic cancer tumors and adjacent normal tissues. **g** Respective IHC staining of NFAT5 and PGK1 in orthotopic xenograft models. **h** Respective IHC staining of NFAT5 and PGK1 in KPC mice. Assays shown in Fig. 5c, d were performed in hypoxia condition. *P < 0.05, **P < 0.01, ****P < 0.0001.

**Fig. 6:**
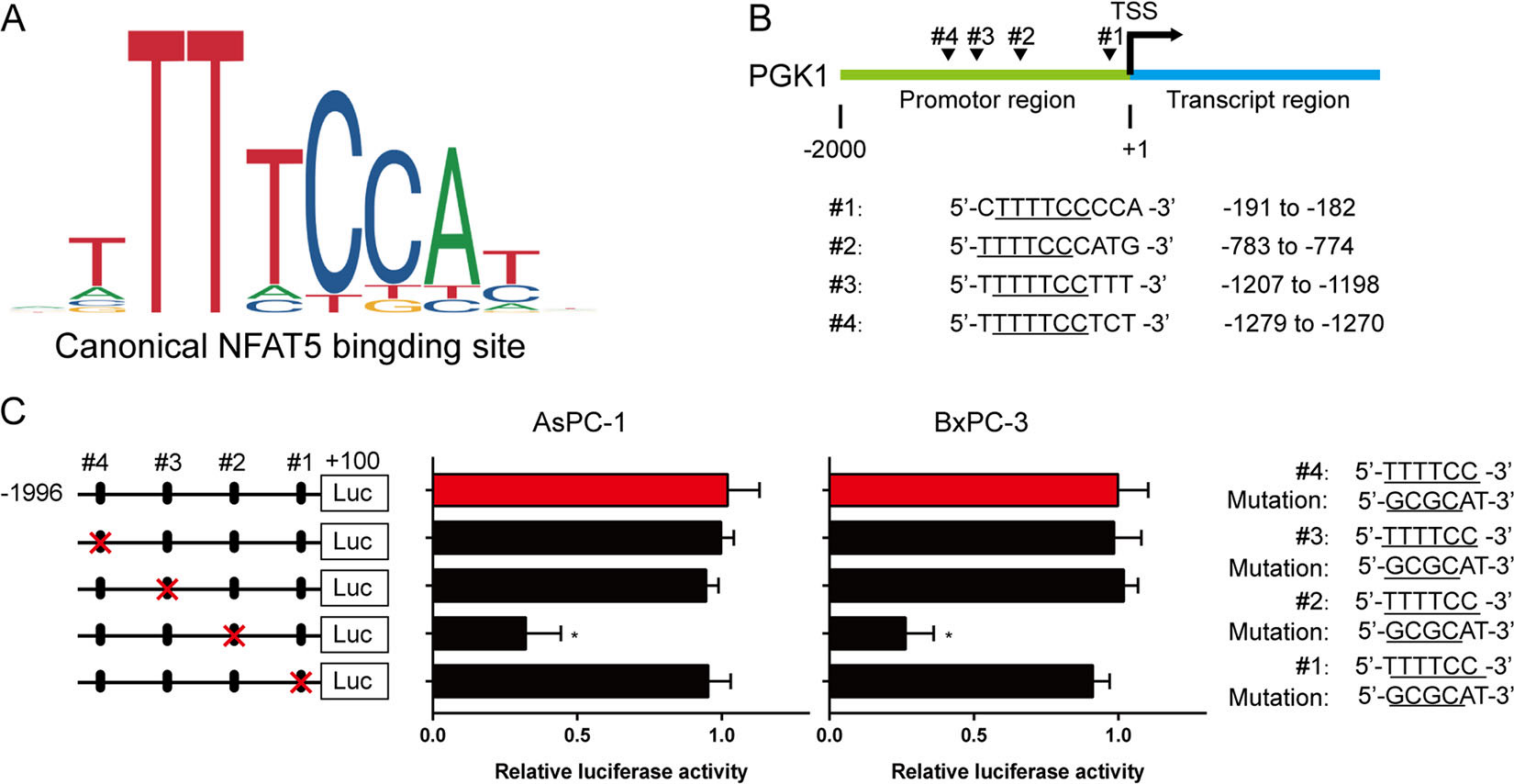
PGK1 is a direct target gene of NFAT5. **a** The sequence logo graph manifested the canonical binding site of NFAT5 predicted by JASPAR. **b** Four potential binding sequences were found in the PGK1 promoter region. **c** Mutated binding sites of NFAT5 lead to impaired luciferase activity in two cell lines. *P < 0.05, **P < 0.01, ****P < 0.0001.

To further understand the underlying mechanism of how NFAT5 contributes to the Warburg effect, as shown in Fig. 5a, several conditions (1) Predicated by the Gene Transcription Regulation Database (GTRD), (2) Differentially expressed in the Renji cohort, (3) Glycolysis marker genes, (4) Top 10% glycolysis-related genes in the Renji cohort, and (5) Significant in prognosis predication) were used to screen for the downstream target genes of NFAT5. MET and PGK1 were then used for subsequent research. We first calculated the correlation between NFAT5 and two target genes in both the Renji cohort and TCGA, which showed that NFAT5 is strongly positively correlated with MET and PGK1 in two datasets (Fig. 5b). We then performed qRT-PCR to further investigate whether knockdown of NFAT5 would decrease the expression of MET or PGK1. As shown in Fig. 5c, d, the expression of PGK1 was significantly decreased in NFAT5 knockdown cell lines, while MET did not exhibit evident differences in different cell lines. To further validate the correlation between PGK1 and NFAT5, we detected the expression of PGK1 in the TMA and found that it was positively correlated with the expression of NFAT5 (Fig. 5e, f. This result was also validated in transgenic models and orthotopic xenograft models (Fig. 5g, h). We then preformed a luciferase assay to investigate whether NFAT5 directly influenced the expression of PGK1. We searched for the canonical binding site of NFAT5 predicted by JASPAR in the promoter region of the PGK1 coding sequence, and four potential binding sites emerged (Fig. 6a, b). After respectively mutating all these four potential binding sites, the luciferase activity of the cells transduced with the third binding site mutant plasmids was significantly lower than that of the other groups in both cell lines (Fig. 6c). Collectively, these data suggest that PGK1 is the direct target gene of NFAT5 in PDAC.

### NFAT5 suppresses proliferation and the Warburg effect by inhibiting PGK1 expression

**Fig. 7:**
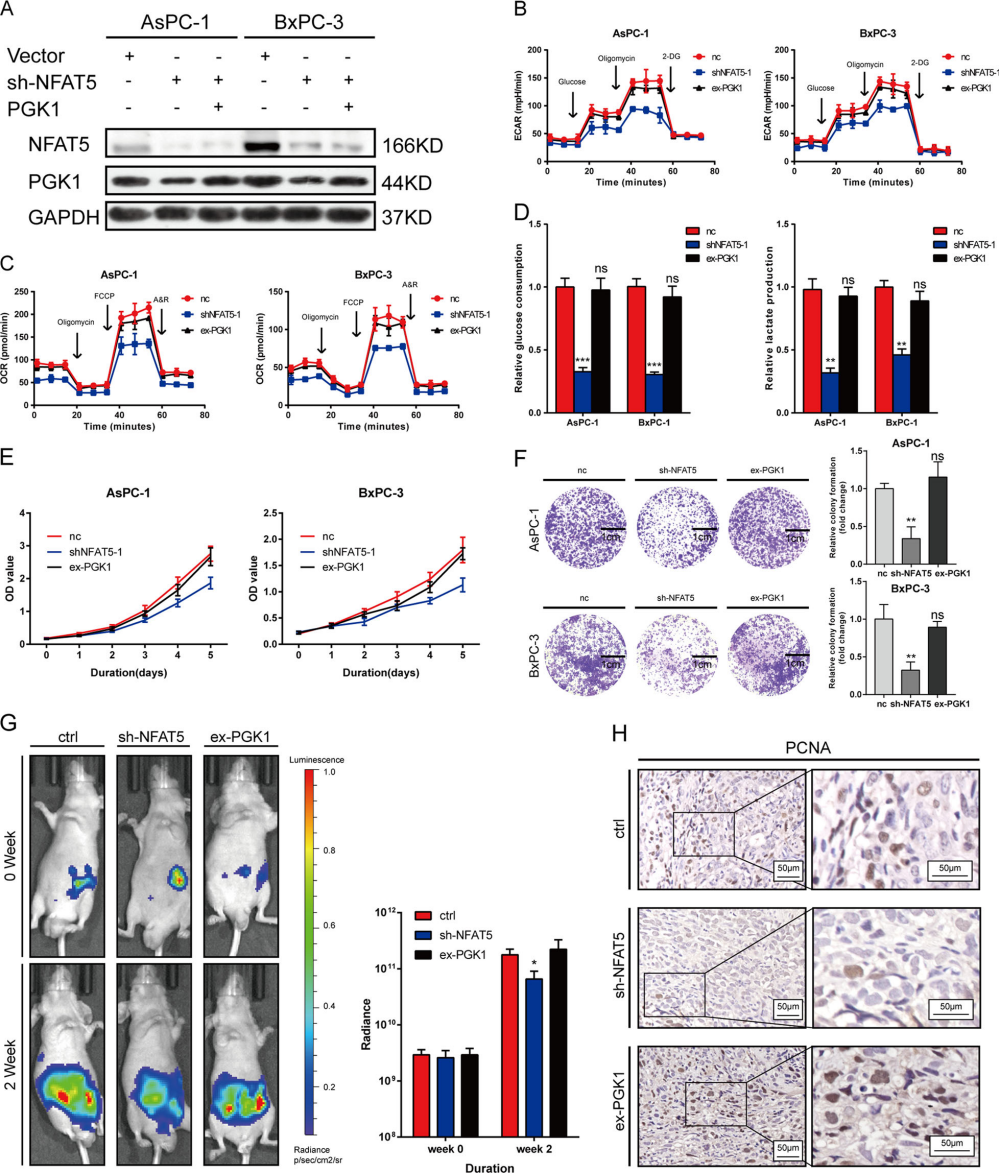
NFAT5 boosts proliferation and the Warburg effect partially via PGK1. **a** Overexpression efficacy of PGK1 in sh-NFAT5-treated AsPC-1 and BxPC-3 cells was determined by western blotting. **b**, **c** Altered level of ECAR and OCR in AsPC-1 and BxPC-3 cells in three different groups (control, shRNA, and PGK1 over-expression). Values are means ± SD. **d** Relative glucose consumption and lactate production in AsPC-1 and BxPC-3 cell lines in three different groups (control, shRNA, and PGK1 over-expression). **e** PGK1-overexpression reversed the inhibitory effects of the knockdown of NFAT5 on the CCK8 assay of PDAC cells, and values are means ± SD. **f** PGK1-overexpression reversed the inhibitory effects of the knockdown of NFAT5 on the colony formation properties of PDAC cells. **g** Representative bioluminescence photograph of mice orthotopically implanted with luciferase-expressing AsPC-1 cells. **h** IHC staining of PCNA in mice orthotopically implanted tissue. Assays shown in Fig. 7b–f were performed in hypoxia condition. Values are means ± SD. *P < 0.05, **P < 0.01, ****P < 0.0001.

To investigate whether NFAT5 performs a pro-cancer function via PGK1, we over-expressed PGK1 in AsPC-1 and BxPC-3 with knockdown of NFAT5. The efficiency was confirmed by WB (Fig. 7a). As revealed by Seahorse ECAR and OCR measurements, expression of PGK1 rescued the Warburg effect in PDAC cells (Fig. 7b, c). Glucose consumption and lactate production assays were performed to validate these results (Fig. 7d). In addition, the pro-survival function of NFAT5 was also compromised with over-expression of PGK1, demonstrated by CCK8 and colony formation assays (Fig. 7e, f). The orthotopic xenograft model was established to test the effect of NFAT5 in vivo. As shown in Fig. 7g, over-expression of PGK1 rescued the decreased bioluminescent emission caused by NFAT5 knockdown. IHC staining of the decreased bioluminescent emission caused by NFAT5 knockdown. IHC staining of PCNA indicated the same result (Fig. 7h).

## Discussion

Since its discovery in the 1920s, the Warburg effect has been widely proven to contribute to malignancy progression and defined as one of the hallmarks of cancer^16^. Via utilizing the Warburg effect, tumor cells generate energy promptly, and the intermediate products of glycolysis are used as proliferation fuel. PDAC has a well-acknowledged mesenchyme-abundant tumor micro-environment (TME), in which the Warburg effect is more crucial for cell survival and proliferation than in a mesenchyme-infertile TME. Therefore, targeting the Warburg effect could be a potential method for PDAC treatment and enhancing other anticancer therapies^14,15,17^.

Here, we demonstrated that transcription factor NFAT5 acts as an oncogene in PDAC through enhancing glycolysis of PDAC cells. The transcription factors of NFAT include NFATC1, NFATC2, NFATC3, NFATC4, and NFAT5, of which only NFAT5 is a significant prognostic factor. Zhou et al. demonstrated that NFAT5 down-regulates mTORC1 signaling in intestinal cells^18^, while we found that NFAT5 is positively correlated with mTOR pathway in PDAC both in vivo and in vitro which is consistent with Hassas’s work^19^. The role of NFAT5 has be extensively studied in the immune system, with little work related to cancer that has been reported^20–22^. In our study, we found that NFAT5 functions as an oncogene in PDAC. Firstly, we showed that the expression of NFAT5 is higher in several datasets both at the protein and mRNA level. Then, we observed that knockdown of NFAT5 leads to a decrease in ECAR and OCR, which further leads to impaired proliferation and colony formation. PET-CT is widely used in clinical diagnosis, in which the 18F-FDG uptake rate is measured and visualized as SUV-MAX. It can reflect the glucose metabolism level of the patient’s tissue. By co-analyzing with IHC results of the same sample, we can assess and validate the influence of different genes on the Warburg effect in clinical PDAC patients. In this study, we showed that with high expression of NFAT5, patients are more likely to have a high SUV-MAX value, which validated our hypothesis that NFAT5 does contribute to the glycolysis of PDAC.

We then set several conditions to find the potential target genes of NFAT5 that affect the glycolysis of PDAC cells. PGK1, a key enzyme in the glycolysis pathway, emerged. PGK1 is the first enzyme generating ATP in glycolysis and is proven to be of vital importance in the progression of several types of cancer^23–25^. In our study, the regulation of PGK1 by NFAT5 was predicted by qRT-PCR, and the binding site was predicted in the promoter region, from which more than four binding sites were found and validated by the luciferase assay. Over-expression of PGK1 was then performed for the rescue assays. We found that the metabolic and proliferation deficiency caused by NFAT5 knockdown were compromised to a certain degree, indicating that PGK1 is one of the major target genes of NFAT5 that leads to PDAC progression.

In conclusion, our work first demonstrated that the expression of NFAT5 is negatively correlated with PDAC patient prognosis. Aberrant high expression of NFAT5 inhibits tumor proliferation and progression by dampening its Warburg effect, which is partly caused by transactivation of PGK1. Targeting the metabolic process of cancer cells may be a hopeful way in tumor treatment^26^. Our work presents novel insight into the pathology of PDAC and a potential target for new therapeutic strategies.

## Supplementary information

Figure S1

Figure S2

Figure S3

Supplementary figure legends
