# Supplementary figures and images for: Transcription factor NFAT5 contributes to the glycolytic phenotype rewiring and pancreatic cancer progression via transcription of PGK1
Source: Cell Death Dis. 2019 Dec 11;10(12):948. doi: 10.1038/s41419-019-2072-5 (PMC6906509; doi:10.1038/s41419-019-2072-5)

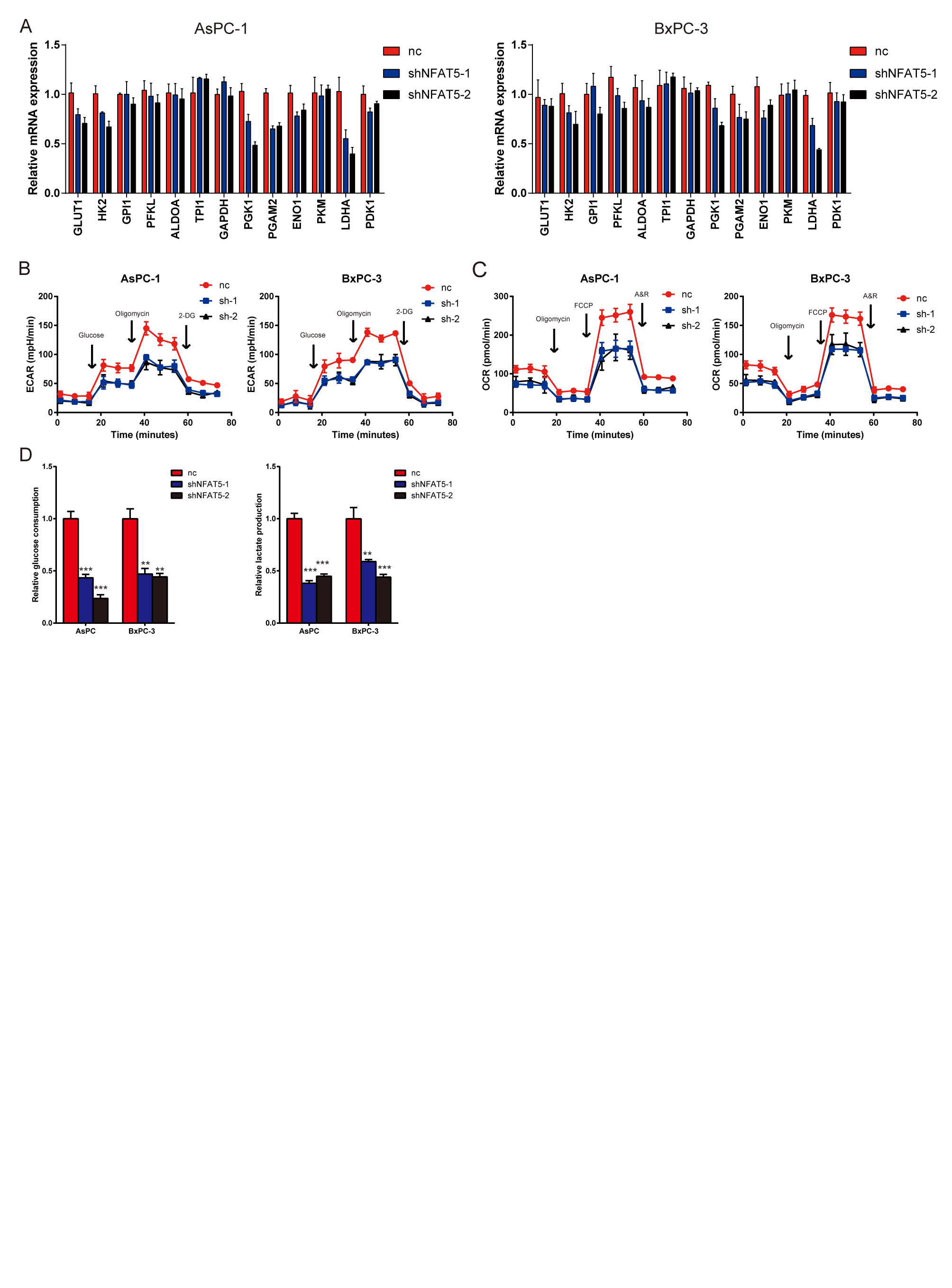

Supplement: Supplementary file 1 — Figure S1 [file 41419_2019_2072_MOESM1_ESM.tif]

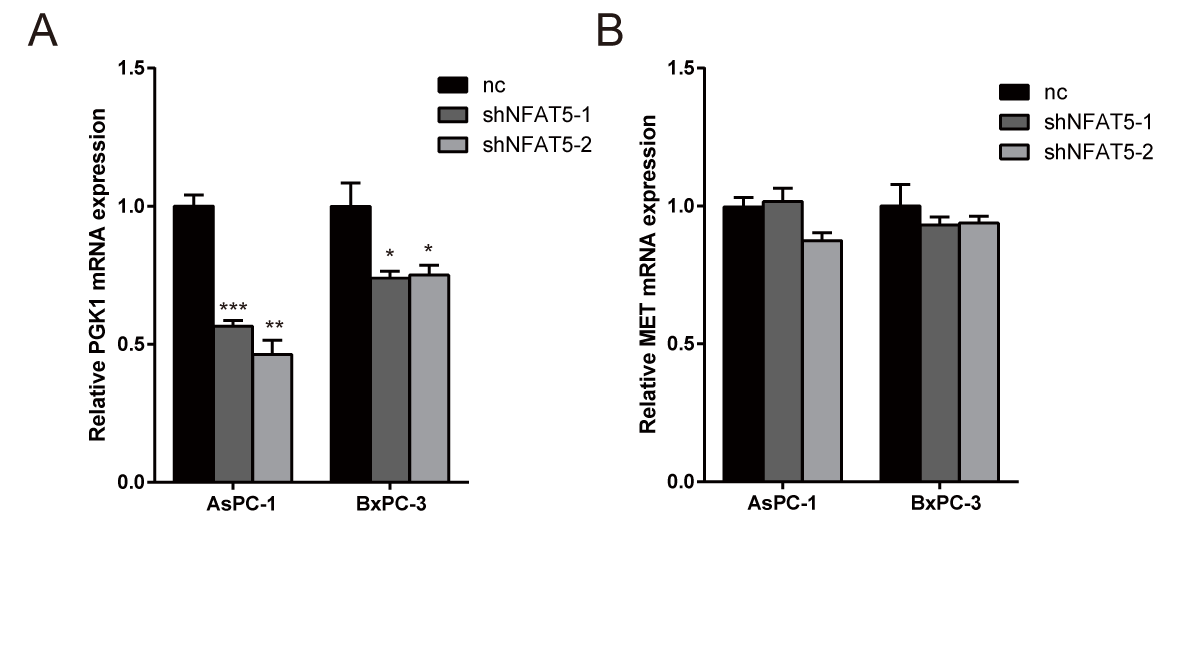

Supplement: Supplementary file 2 — Figure S2 [file 41419_2019_2072_MOESM2_ESM.tif]

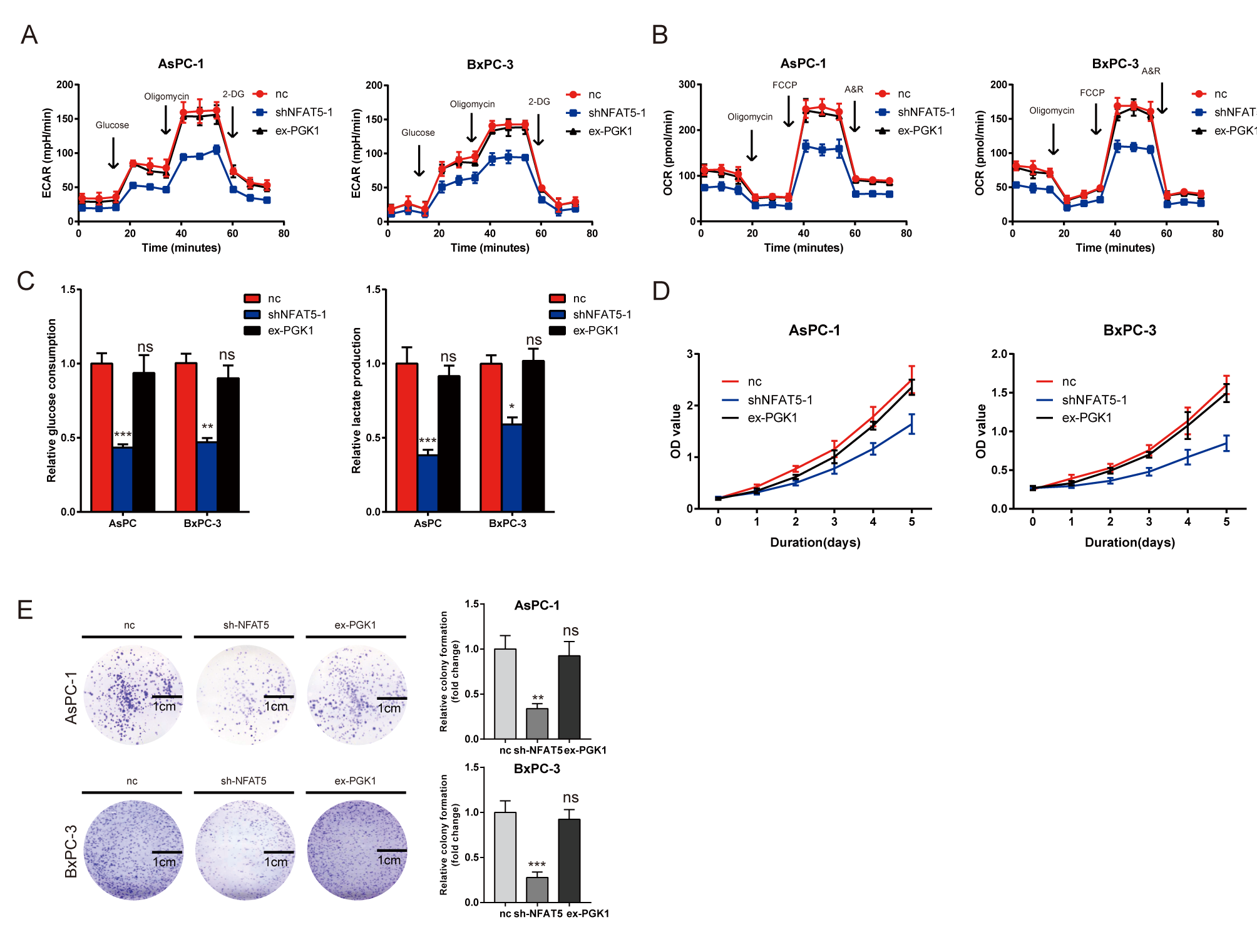

Supplement: Supplementary file 3 — Figure S3 [file 41419_2019_2072_MOESM3_ESM.tif]
