# Supplementary material for: Transcription factor NFAT5 contributes to the glycolytic phenotype rewiring and pancreatic cancer progression via transcription of PGK1
Source: Cell Death Dis. 2019 Dec 11;10(12):948. doi: 10.1038/s41419-019-2072-5 (PMC6906509; doi:10.1038/s41419-019-2072-5)
Supplement: Supplementary file 4 — Supplementary figure legends [file 41419_2019_2072_MOESM4_ESM.docx]

**Supplementary Figure legend**

Fig. S1 (A) Relative mRNA levels of glycolysis-related genes of PDAC with or without NFAT5 knockdown. (B-C) Glycolytic function and mitochondrial stress test of NFAT5 knockdown AsPC-1 and BxPC-3 cells treated with shRNA were measured by extracellular acidification rate (B) and oxygen consumption rate (C), respectively. (D) Relative glucose consumption and lactate production in normal and NFAT5 knockdown AsPC-1 and BxPC-3 cell lines.

Fig. S2 (A-B) Relative PGK1 and MET mRNA expression in the control and NFAT5 knockdown PDAC cell lines.

Fig. S3 (A-B) Altered level of ECAR and OCR in AsPC-1 and BxPC-3 cells in three different groups (control, shRNA, and PGK1 over-expression). Values are means ± SD. (C) Relative glucose consumption and lactate production in AsPC-1 and BxPC-3 cell lines in three different groups (control, shRNA, and PGK1 over-expression). (D) PGK1-overexpression reversed the inhibitory effects of the knockdown of NFAT5 on the CCK8 assay of PDAC cells, and values are means ± SD. (E) PGK1-overexpression reversed the inhibitory effects of the knockdown of NFAT5 on the colony formation properties of PDAC cells.
